# Influence of Chemical Pre-Treatments and Ultrasonication on the Dimensions and Appearance of Cellulose Fibers

**DOI:** 10.3390/ma13225274

**Published:** 2020-11-21

**Authors:** Bartłomiej Mazela, Waldemar Perdoch, Barbara Peplińska, Mikołaj Zieliński

**Affiliations:** 1 Faculty of Forestry and Wood Technology, Poznan University of Life Sciences, Wojska Polskiego 28, 60-637 Poznan, Poland; waldemar.perdoch@up.poznan.pl (W.P.); zielinski.mikolaj@vp.pl (M.Z.); 2 NanoBioMedical Centre, Adam Mickiewicz University, Umultowska 85, 61-614 Poznań, Poland; barp@amu.edu.pl

**Keywords:** cellulose, MCC, nanocellulose, CNC, CNF, ultrasonication, TEMPO, SEM

## Abstract

Due to the wider use of nanocellulose in various areas of economic life, better and more optimal methods of obtaining nanocellulose are constantly being sought. Therefore, an attempt was made to evaluate the hybrid cellulose treatment, based on the use of a chemical method combined with an ultrasound of medium frequency. The study employs two different starting materials (Södra Black R cellulose or microcrystalline cellulose), two types of chemical pre-treatments (acid hydrolysis or oxidation), and two sonication durations. It was found that the reduction fiber cross-sectional dimensions was the result of prolonged exposure of cellulose to the ultrasound. From Södra Black R and the microcrystalline cellulose nanometer scale, structures were obtained in the form of isolated fibers. The TEMPO reagent accelerated the degradation process of two cellulose varieties due to its oxidizing character. The resulting products had nanofibrous structures. Cellulose degradation as a result of the combined action of sonication and TEMPO activity progressed gradually. Places of fiber degradation were characterized by their longitudinal breakage and initiated the next stages of the defibering process.

## 1. Introduction

**Figure 1:**
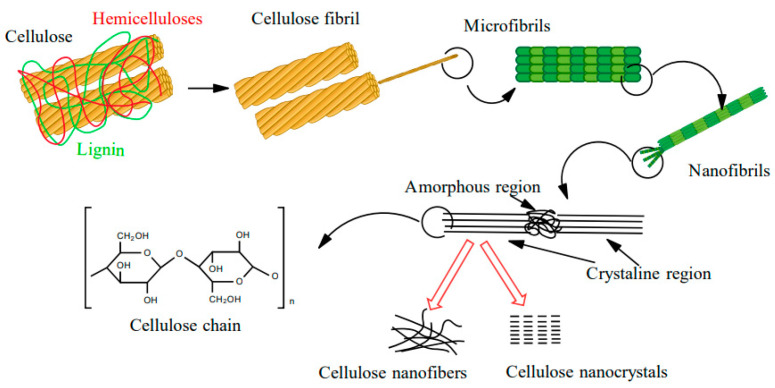
Cellulose structures.

Interest in nanotechnology has increased significantly in recent decades, although the first research reports on the subject of nanoparticles were published in the 1950s [1]. This rapidly-developing field of science is increasingly focused on issues related to nanomaterial fabrication, properties, and the use of nanoparticles with natural origins, including nanocellulose, which is often considered the “material of the future” [2]. Cellulose is a biopolymer commonly found in nature, e.g., in plants, bacteria, or algae [3]. The structural units of cellulose molecules are D-anhydro glucopyranose, which are linked by a β-1,4-glycosidic bond. In nature, cellulose typically fulfills a skeletal function in plant cell walls and exists there in the form of macrofibrils. Macrofibrils are made of microfibrils, which are, in turn, composed of nanofibrils. The lattermost are crystalline or amorphous nanoparticles. The crystalline regions of cellulose are strongly linked through hydrogen bonds between hydroxyl groups, thus making them difficult to separate (Figure 1).

Nanocellulose (NC) is a biopolymer characterized by an organized structure, in which at least one cross-sectional dimension is on the nanometer scale, i.e., less than 100 nm. Three types of NC can be distinguished, namely nanocrystalline cellulose (CNC), nanofibrous cellulose (CNF), and bacterial cellulose (BC) [4]. CNC, which is most commonly extracted from cellulose fibers via acid hydrolysis, resembles elongated crystalline rods, with a compact ordered structure that enables less elasticity than the structure of CNF [5,6,7]. This material exhibits a low proportionality factor, and the diameter and length of a single “rod” varies from 2 to 20 nm and from 100 nm to several µm, respectively [8,9]. The degree of crystallinity of CNC is typically between 54–88% [10]. Habibi et al. [11] emphasized that the degree of crystallization, size distribution, and morphology depend on the starting material and the conditions by which the NC was obtained. In contrast, CNF consists of bundles of cellulose chains [12] bound into long, flexible, tangled nanofibers ranging from 1–100 nm in length [13], and they represent the smallest plant fiber structures. Similar to the macromolecular cellulose, CNF consists of alternating crystalline and amorphous regions [5] and it is usually formed as a result of mechanical, chemical, or enzymatic treatment [14].

NC can be fabricated using either a “bottom-up” or a “top-down” method. Bacterial nanocellulose is synthesized by the “bottom-up” method (i.e., forming larger units from smaller building blocks) during the fermentation of low-molecular weight sugars, with the assistance of *Acetobacter* bacteria. In contrast, “top-down” methods focus on disintegrating natural fibers into smaller units through mechanical and/or chemical treatment [15,16]. The most prominent methods for obtaining NC by a “top-down” approach are mechanical (e.g., high-pressure homogenization, microfluidization, milling, cryogenic milling, or high-intensity ultrasonication), enzymatic, and chemical (e.g., using ionic liquids, acid-base treatment, acid hydrolysis, or oxidation).

High-intensity ultrasonication (HIUS) is one type of mechanical approach for fabricating NC. This treatment method involves separating the fibers from the cell wall using cavitation in an aqueous environment. When water molecules absorb the applied ultrasonic energy, microscopic gas bubbles form, grow, and implode on the surface of the cellulose. The resulting hydrodynamic forces cause defibrillation of the starting material, which may be pure cellulose, microcrystalline cellulose (MCC), pulp, banana peel, rice straw, or microfiber cellulose, etc. Studies have shown that the mixture of nano- and microfibrils obtained after an ultrasonic bath can reach dimensions of 20 nm up to several micrometers, which indicates that the product mixture contains some nanofibers which are isolable and some that are not separated from microfibrils. This method enables the production of filament aggregates with varying sizes. Depending on the characteristics of the starting material, an ultrasonic bath will affect the crystalline structure of the cellulose in different ways. For example, in the case of pure cellulose, the degree of crystallinity increased following the treatment, whereas in the case of MCC, the degree of crystallinity decreased, and in the case of the cellulose pulp, the degree of crystallinity remained constant [17]. Wang and Cheng [18] described how ultrasonic processing parameters impact the degree of cellulose defibrillation. They demonstrated that the degree of defibrillation increased with increases in both the device power and the process temperature. Additionally, a more concentrated cellulose suspension and a greater distance between the working vessel and the ultrasonic probe did not improve the defibrillation process. It was also determined that longer fibers were less prone to defibrillation than shorter ones. In addition, the combined use of HIUS and high-pressure homogenization (HPH) methods enhanced the defibrillation efficiency. Another combined approach paired HIUS with oxidation by the 2,2,6,6-tetramethylpiperidine-1-oxyl reagent (TEMPO), and achieved an NC yield of 71% [19]. Alternatively, milling a sample of TEMPO pre-treated cellulose pulp produced NC in 90% yield. Oxidation with the TEMPO reagent also led to an additional increase in the NC yield obtained using the ultrasonic probe (100% efficiency) and the ultrasonic bath (50% efficiency). Chen et al. [20] showed that the HIUS treatment of bamboo, wood, and wheat straw generated NC with increased cellulose crystallinity (60%) and a degradation temperature over 330 °C.

**Figure 2:**
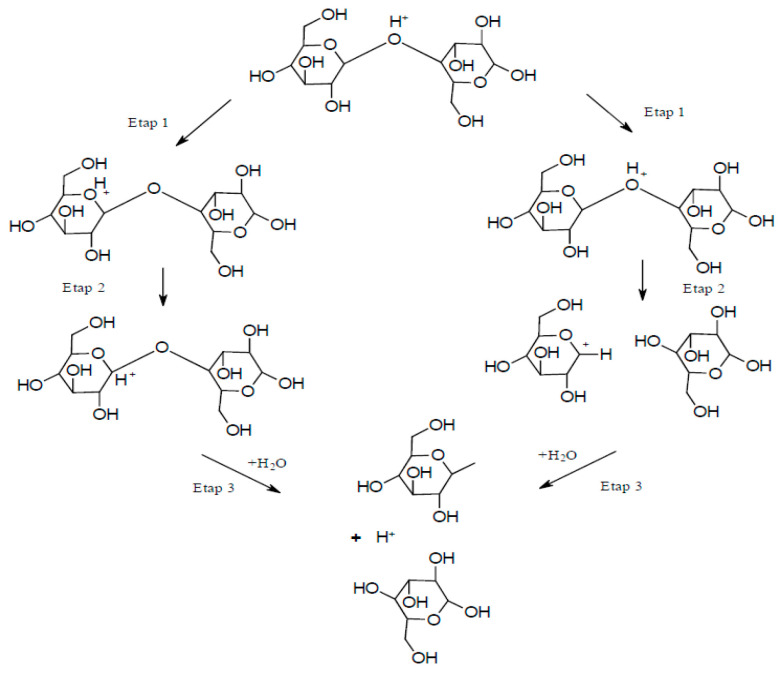
Acid hydrolysis process.

Although chemical techniques represent direct methods for extracting NC, they can also be applied in intermediate stages, such as during pre-treatment to promote further refinement of the raw material. The goal of implementing such intermediate stages is primarily to reduce the energy consumption of the mechanical processes used in the downstream stages of NC fabrication. In fact, the high energy consumption is a major disadvantage related to the process of obtaining NC. Applying a pre-treatment reduces the energy requirement from an average of 25,000 to 1000 kWh/ton of cellulose fiber [21]. The pre-treatment usually involves treating cellulose with enzymes, ionic liquids, acids, or alkaline reagents, or subjecting cellulose to oxidation reactions (e.g., in the presence of TEMPO). The alkaline-acid treatment was most commonly used as a pre-treatment prior to a mechanical treatment in the three-step process of obtaining CNF [6,22,23]. The first step of this method consisted of soaking the fibers in a solution of sodium hydroxide to hydrolyze them. The second step involved hydrolysis of the fibers using hydrochloride to dissolve hemicellulose, and the third step employed sodium hydroxide to break down the structure of the lignin and cleave the bonds between carbohydrates and lignin. Using such an alkaline-acid pre-treatment scheme, NC can be extracted from wheat straw and soybean husks [23]. The resulting fibrous material was characterized as NC, with cellulose fiber dimensions (on the cross-section) of 10–80 and 20–120 nm, respectively. Acid hydrolysis is the oldest method for obtaining CNC. The hydrolysis reaction rate depends on the temperature and the concentration of the acid used. During hydrolysis, amorphous areas decay and crystalline areas remain intact. The hydrolysis mechanism can be divided into three steps [24]: (i) Proton binding to an oxygen atom of a glycosidic bond or an oxygen atom between two carbon atoms in an anhydro glucopyranose ring, (ii) proton transfer to the C1 atom leading to the formation of a carbocation and cleavage of the glycosidic bond, and (iii) release of simple sugars and regeneration of the proton due to the interaction between water and the carbocation (Figure 2).

**Figure 3:**
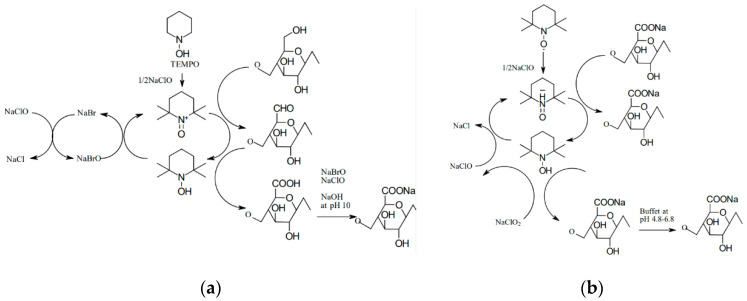
Mechanism of oxidation using the TEMPO reagent (**a**) in alkaline solution and (**b**) in acidic solution.

The so-called controlled hydrolysis is carried out in such a way to promote the formation of shortened polymer chains rather than a complete destruction of cellulose into simple sugars. A 60–65% aqueous solution of sulfuric acid is most commonly used for this purpose, and the reaction is conducted at 45–75 °C over a period of 40–70 min. A disadvantage of this method is that sulfone groups tend to persist in the resulting CNC, which leads to a low thermal stability. However, other acids, such as hydrochloric acid or hydrobromic acid, can be used, but they typically produce NC with a higher tendency to agglomerate. Combining acid and ultrasonic treatments led to a more than 20% increase in crystallinity relative to the starting material. The oxidation reaction with TEMPO in an aqueous solution has also been described extensively in the literature. During this reaction, secondary hydroxyl groups remain intact, while a carboxyl or aldehyde group is formed with a negative charge at the C6 position. The reaction can be carried out at room temperature and in an alkaline solution in the presence of NaClO, NaBr, and catalytic amounts of the TEMPO reagent [25]. It was discovered that when the process is conducted in an alkaline solution, aldehyde groups are formed, resulting in reduced thermal stability, hindered microfibrillation, and discoloration of the NC after drying (Figure 3a). In contrast, conducting such a process in an acidic environment (pH 6.0–6.5) at a temperature of 50–60 °C increases the product’s thermal stability (Figure 3b). In this case, no aldehyde groups are formed, and consequently, there is no uncontrolled cellulose depolymerization [17,25].

Liew et al. [26] described the results of acid hydrolysis of cellulose (i.e., native cellulose) yielding CNC I. It is clear from the performed microimaging that the cellulose has been defibrillated and the size of its particles has been reduced. The authors observed the fragmentation of particles during preparation of CNC II from cellulose II (i.e., cellulose obtained by mercerization and regeneration of native cellulose). The particles were characterized by irregular shapes close to the spherical [27,28]. Kim et al. [2] observed that the structure of nanocellulose fibers largely depends on their origin. This conclusion arose from comparing the structure of BC, MCC, and bamboo cellulose fibers.

In light of the literature analysis presented here, this work aims to evaluate the effects of hybrid cellulose treatments, specifically, chemical methods paired with medium frequency ultrasonication. The study employs two different starting materials (Södra Black R cellulose or microcrystalline cellulose), two types of chemical pre-treatments (acid hydrolysis or oxidation), and two sonication durations at 45 kHz (1 or 6 h). The NC products are characterized using scanning electron microscopy, and the impacts of treatment parameters are discussed.

## 2. Materials and Methods

### 2.1. Materials

Cellulose in the commercial form of Södra Black R and microcrystalline cellulose (Sigma-Aldrich, Darmstadt, Germany) were used as the starting materials for nanocellulose generation. The Södra Black R cellulose was a mixture of wood fibers from *Picea abies* (80%) and *Pinus sylvestris* (20%) and had the following performance parameters: Average fiber length (2050 µm); average fiber width (30.0 µm); coarseness (0.135 µg/m); brightness (89.5%); pH (4.8); and ash content (0.2%). The Södra Black R cellulose sheet had a very compact structure. Therefore, it was ground in a laboratory grinder (CHemLand, Stargard Szczecinski, Poland) and then fractionated using a 400 µm sieve to produce a homogeneous raw material.

### 2.2. Hydrolysis of Cellulose

The ground Södra Black R cellulose and microcrystalline cellulose (Sigma-Aldrich) were acid-treated using 65% sulphuric acid (Poch, CAS no. 7664-93-9, Gliwice, Poland) with a mass ratio of cellulose material to acid solution of 1:30. The fibers were dispersed in the acid by mixing with a magnetic stirrer (600 rpm) at 45 °C for 30 min. The stirring time was measured from the moment the desired process temperature was reached. To complete the reaction and neutralize the pulp, the raw material was rinsed 6 times with 100 mL of distilled water. The washed raw material was centrifuged using a laboratory centrifuge (2500 rpm, 20 min, Scilogex DMO412 Rocky Hill, CT, USA) and then decanted with the sediment solution (,. The treated pulp was dried in a laboratory dryer at 40 °C.

### 2.3. Cellulose Oxidation

The treatment with TEMPO (Pol-Aura, CAS no. 2564-83-2, Gliwice, Poland) was carried out by introducing cellulose into a saturated solution of the TEMPO reagent (9.7 g/dm^3^). In order to increase the pH of the solution (to pH > 8), the NaOH solution (Poch, CAS no. 1310-73-2, Gliwice, Poland) was added to the reaction mixture in a proportion of 0.1 g per 9 mL. The oxidation process took place during sonication in the ultrasonic cleaner (POLSONIC Palczyński Sp. J., Warszawa, Poland).

### 2.4. Cellulose Sonication

Sonication was carried out in tightly-closed 15 mL vials. Suspensions with a cellulose concentration of 1% (0.09 g of cellulose material per 9 g of liquid) were treated in a 45 kHz ultrasonic cleaner. The raw material was (i) placed in distilled water when no pre-treatment or acid treatment was involved, or (ii) placed in an alkaline solution saturated with TEMPO, depending on the desired experiment. The vials were placed in a POLSONIC SONIC-6D ultrasonic cleaner and subjected to sonication for a designated time period: Either for 60 min or for two cycles of 180 min each (i.e., 6 h total). The initial temperature of the bath was 18 °C, but during the process, it increased significantly (rising to approx. 60 °C after 3 h). In order to avoid excessive heating of the system, after the first 3 h of sonication, the process was interrupted for 24 h before continuing for the final 3 h.

### 2.5. SEM Analysis

Direct observation of the microstructure morphology of cellulose before and after the chemical and mechanical treatment was examined by the SEM method. The samples were placed on a double sided adhesive carbon tape and coated with a gold layer of thickness of 10 nm. Images were taken with the use of JEOL JSM-7001F TTLS (JEOL Ltd., Tokio, Japan) scanning electron microscope applying the accelerating voltage of 5 kV, a working distance of about 10 mm (depending on the focus), and a secondary electron (SEI) detector. The sizes of cellulose fibers were manually measured using ImageJ^®^ (an open source Java image processing program inspired by NIH Image) from 20 particles shown in three independent images using ImageJ^®^1.52v.

## 3. Results

### 3.1. Microscopic Analysis of Södra Black R Cellulose

**Figure 4:**
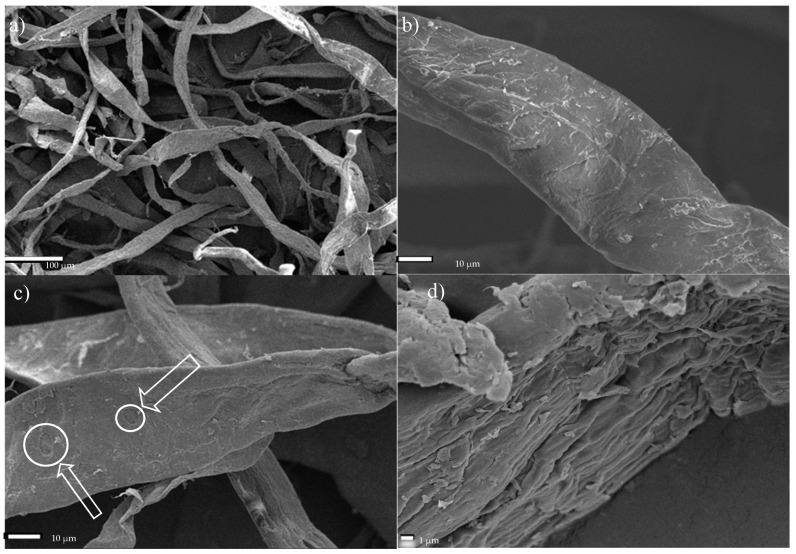
SEM micrographs of Södra Black R cellulose fibers. (**a**) Fiber network, (**b**) single fiber, (**c**) fibers with degraded areas—characteristic oval shapes as a starting point of degradation, (**d**) microfibril arrangement.

Microscopic images of the fiber network (Figure 4a) and a single cellulose fiber of Södra Black R, which has undergone a mechanical pre-treatment (Figure 4b), were obtained using SEM. The cross-sectional dimensions of the fibers ranged from 22.5–43.8 µm, with an average value of 32.95 ± 8.2 µm. In some samples (Figure 4c), it was possible to identify areas with more degraded structures near the central part of the fibers, which had characteristic oval shapes. Further analysis revealed that these areas represented the beginning of the subsequent division of the fiber into smaller parts. Figure 4d presents a cellulose fiber observed under 4000× magnification, wherein individual fibers consisting of parallel microfibrils are visible.

**Figure 5:**
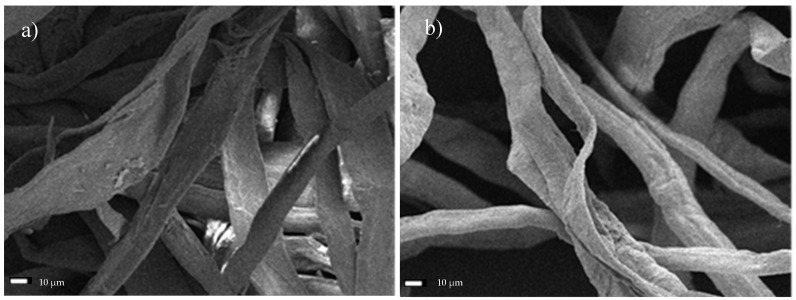
SEM micrographs of Södra Black R cellulose subjected to the ultrasound for a period of (**a**) 1 h, or (**b**) 6 h.

**Figure 6:**
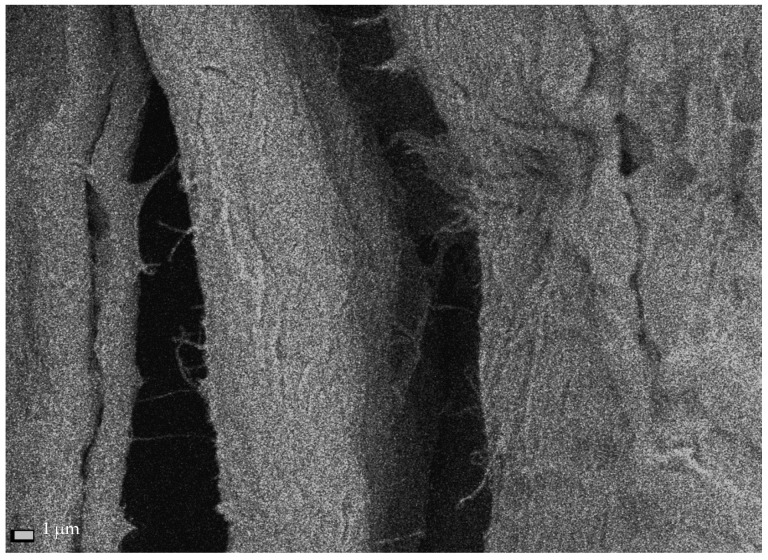
Single nanofibrils extracted from Södra Black R cellulose fibers after 6 h of sonication.

The cross-sectional dimensions of Södra Black R cellulose fibers subjected to the ultrasonic treatment for 1 h ranged from 15.4–37.4 µm, with an average dimension of 23.58 ± 8.2 µm (Figure 5a). Therefore, the ultrasound reduced the size of the fibers by approx. 28%, relative to the starting material. After the 6-h sonication process, the transverse dimensions of the cellulose fibers ranged from 11.9–36.4 µm, and their average value was 20.31 ± 7.3 µm (Figure 5b). In this case, the average fiber dimensions decreased by approx. 38%, relative to the dimensions of the initial material. The areas where the initial nanofibers began to separate could be identified on the measured sample (Figure 6). This figure shows single fibers with much smaller cross-sections separating from the cellulose fiber.

### 3.2. Dual Treatment of Södra Black R Cellulose with Ultrasonication and either TEMPO or Acid Hydrolysis

**Figure 7:**
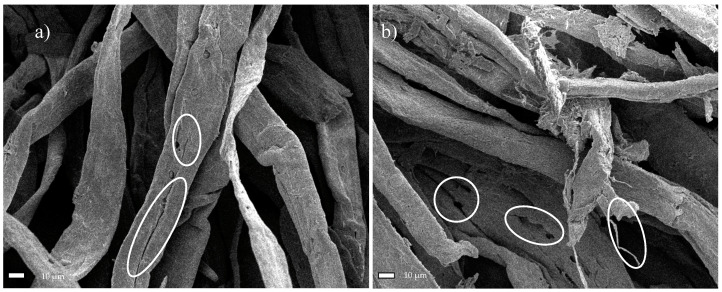
SEM micrographs of Södra Black R after oxidation with TEMPO and the ultrasonic treatment (with marked characteristic oval shapes as a starting point of degradation) for (**a**) 1 h, or (**b**) 6 h.

**Figure 8:**
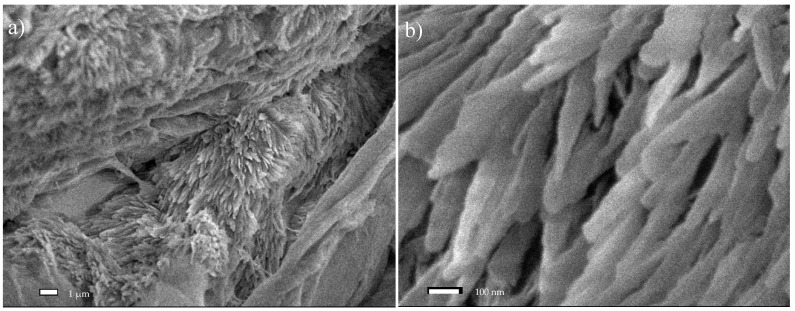
SEM micrographs of Södra Black R cellulose after treatment with TEMPO as an oxidizer and ultrasound: An image of a cracking fiber (**a**) ×10,000; (**b**) ×90,000.

After 1 h of ultrasonic treatment with the TEMPO reagent, the fibers had dimensions in the range of 19.5–32.8 µm, with a mean value of 25.97 ± 5.2 µm. This corresponds to an approx. 21% decrease in cross-sectional fiber dimensions, relative to the starting material. Figure 7a shows the deepening of oval cavities on the fibers’ surface, resulting in holes and cracks along the longer dimension. In Figure 7b, nanometer scale structures were observed at the site of a parallel crack along the fiber’s length. The oxidation and sonication process conducted over a period of 6 h resulted in defragmentation of the cellulose fibers, and their transverse dimensions ranged from 6.5–22.9 µm. The average measured transverse dimension of the fibers was 13.77 ± 5.2 µm. In this case, the fibers displayed an approx. 58% reduction in size, relative to the raw material. Figure 8a,b shows the area where the fiber was broken by oxidation and sonication. At this large magnification (90,000×), numerous nanometer-sized regions can be observed.

**Figure 9:**
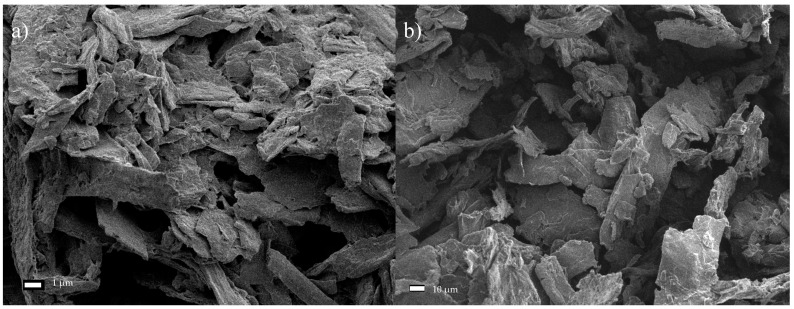
SEM micrographs of the Södra Black R cellulose after subjecting them to acid hydrolysis and ultrasounds for a period of (**a**) 1 h, or (**b**) 6 h.

Figure 9a shows a microscopic image of Södra Black R cellulose that has been subjected to acid hydrolysis and then sonication for a period of 1 h. The size range of the resulting particles was between 5.1 and 42.3 µm, and the average cross-sectional dimension of the tested cellulose fibers was 16.7 ± 10.3 µm. Therefore, the dimensions were reduced by approx. 49% on average, relative to the starting material. An image depicting cellulose after acid hydrolysis, followed by sonication for 6 h is presented in Figure 9b. The size range of these modified cellulose fibers was between 3.4 and 42.2 µm, and the average cross-sectional dimension was 19.28 ± 12.6 µm. These values correspond to an approx. 41% percentage decrease in dimensions, relative to the original material. Following the acid treatment, the raw material lost its fibrous structure and formed agglomerates of irregular particles. It should be noted that during the sample preparation for analysis (i.e., during drying), a change in the color of the cellulose was observed in some areas. This was most likely due to the attachment of sulfone groups to the cellulose structure, which significantly reduced the thermal stability of the material [28].

### 3.3. Microscopic Analysis of Microcrystalline Cellulose

**Figure 10:**
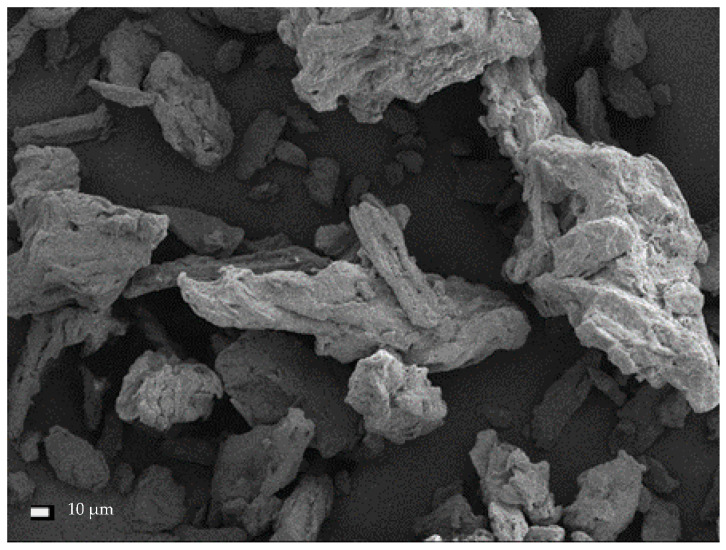
SEM micrographs of untreated microcrystalline cellulose (MCC).

The average dimensions of microcrystalline cellulose ranged from 25.4–97.2 µm, and the average size was 51.79 ± 22.7 µm (Figure 10). This cellulose took the form of irregular lumps (crystals).

**Figure 11:**
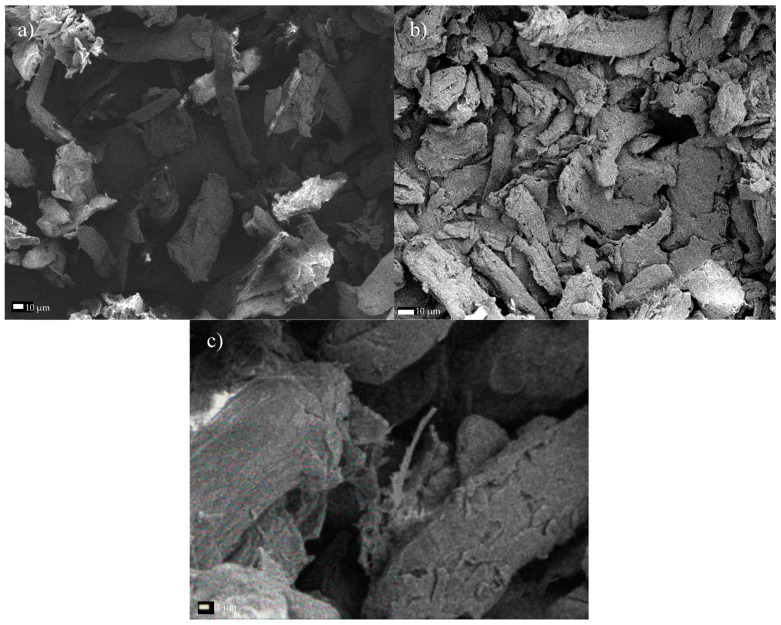
SEM micrographs of microcrystalline cellulose subjected to sonication for a period of (**a**) 1 h, or (**b**) 6 h, and (**c**) the filament separating after 6 h of treatment.

When subjected to the ultrasonic treatment for a period of 1 h, the cross-sectional dimensions of the cellulose crystals generally decreased and were within the range from 12.1–53.1 µm. The mean dimension was 29.21 ± 15.1 µm (Figure 11a), representing an average decrease in dimensions of 44% compared with untreated MCC. Further defragmentation of the cellulose was observed with the longer sonication time (6 h; Figure 11b), yielding particles measuring 11.1–25.5 µm (with a mean value of 17.62 ± 4.6 µm). The decrease in dimensions relative to the starting material corresponded to 66%. The image of the test material captured with a higher magnification (Figure 11c) enabled the identification of the effect of isolating the cellulose nanofibers. The image clearly shows the filament breaking off, with cross-sectional dimensions in the 890 nm range. Further micro- and nanofibers were also observed to be detaching from the MCC fibers.

### 3.4. Dual Treatment of MCC with Ultrasonication and either TEMPO or Acid Hydrolysis

**Figure 12:**
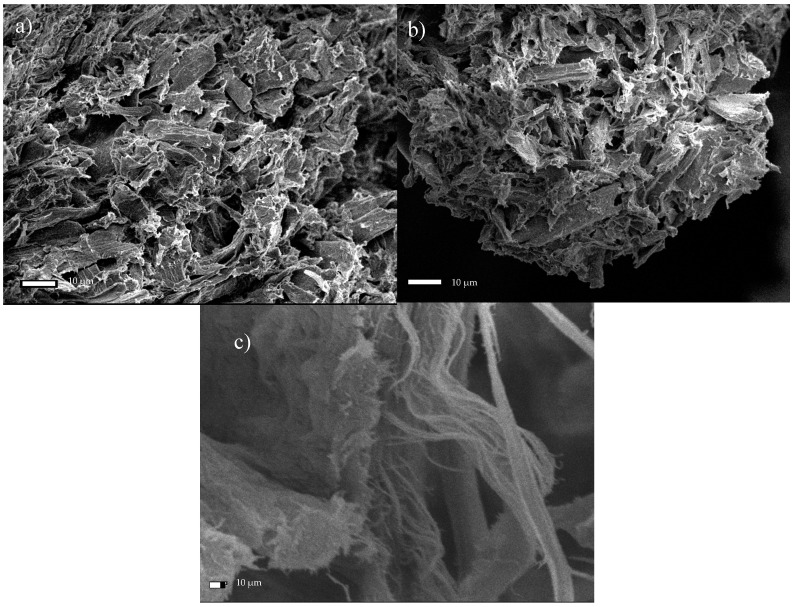
SEM micrographs of microcrystalline cellulose subjected to sonication in the presence of the TEMPO reagent for a period of (**a**) 1 h, or (**b**) 6 h, and (**c**) an image of the isolated fiber after 6 h of exposure.

After treating the microcrystalline cellulose with 1 h of ultrasonic treatment and oxidation in the presence of the TEMPO reagent, the dimensions of the resulting product decreased by approx. 90%, relative to the original substrate. The average cross-sectional dimension of the MCC fibrous products was 5.06 ± 2.6 µm, and the individual dimensions ranged from 1.9–9.3 µm (Figure 12a). After 6 h of sonication in the presence of TEMPO, cellulose particles with dimensions ranging from 1.9–8.7 µm were obtained, where the average fiber dimension was 5.17 ± 3.0 µm (Figure 12b). Therefore, no significant additional changes were observed in the modified cellulose structures subjected to the prolonged (6 h) treatment. In Figure 12c, the defibrillation effect is clear, which allowed nanometric fibers to be isolated. Fibrous structures with dimensions in the range of 8–120 nm are clearly visible in the studied structure.

**Figure 13:**
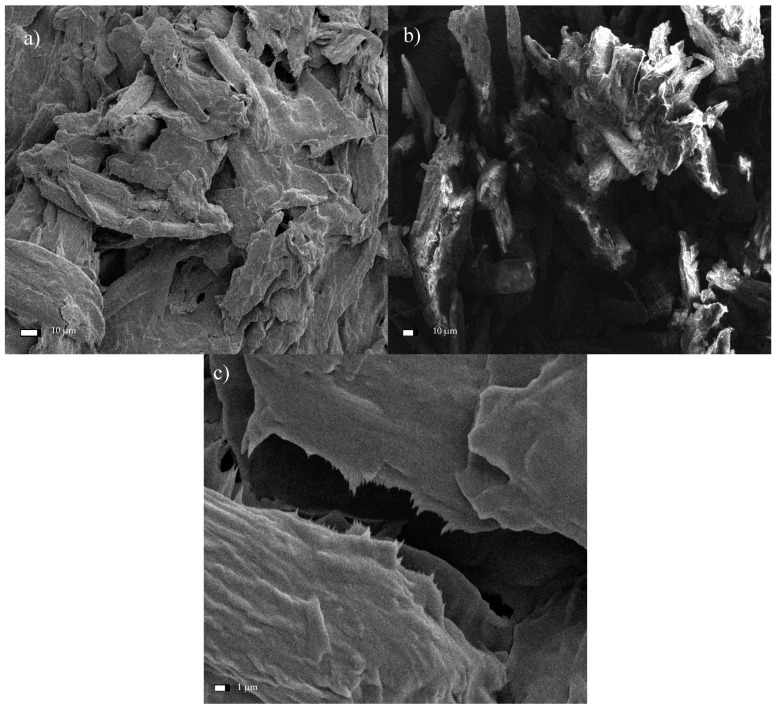
SEM micrographs of the microcrystalline cellulose hydrolyzed and then sonicated for a period of (**a**) 1 h, or (**b**) 6 h, and (**c**) an image of a fiber fracture after 1 h of exposure.

As with the Södra Black R cellulose, MCC was heavily degraded during a pre-treatment involving acid hydrolysis. After hydrolysis and subsequent 1 h of sonication, the MCC dimensions were approx. 65% smaller than in the unmodified MCC. In Figure 13a, areas where the sizes of cellulose particles ranged from 6.8–39.2 µm (with a mean dimension of 17.15 ± 10.4 µm) are observed. It was also observed that the particles (lumps, in this case) of hydrolyzed and sonicated cellulose were connected to each other (Figure 13), whereas the unmodified MCC particles did not interact with each other (Figure 10). As a result of increasing the sonication time from 1 to 6 h, cellulose fibers showed a tendency to agglomerate. As a consequence, their cross-sectional dimension increased twice. The average particle size of the cellulose in this case was 31.23 ± 15.2 µm (Figure 13b). This phenomenon can be explained by a greater tendency of degraded material to agglomerate into larger particles over time under these conditions. Figure 13c presents an image depicting an area where the cellulose fiber further degraded into smaller fragments.

## 4. Conclusions

The performed experiments on the hydrolysis and oxidation of commercial and microcrystalline cellulose, combined with its exposure to the ultrasound, allowed assessing the structure of the obtained material through SEM analysis. First, it was found that the reduced fiber cross-sectional dimensions was the result of increased exposure of cellulose to the ultrasound. Specifically, subjecting both Södra Black R cellulose and MCC to the ultrasound for 6 h led to nanometer scale structures in the form of isolated fibers. Second, the TEMPO reagent accelerated the degradation process of the two cellulose varieties due to its oxidizing character. The resulting products had nanofibrous structures. Third, the degradation of cellulose through the combined action of sonication and TEMPO activity was gradual. Early effects of the treatment manifested in characteristic oval regions, where the initiation of surface fiber degradation took place. In the next stage, these areas showed further progression of longitudinal cracking in the fibers. Additionally, a significant degradation of the cellulose structure, creating a solid/crystalline structure from the fibrous system, occurred as a result of the acid treatment. The degradation of cellulose continued throughout the process of sonication, and increasing time led to the agglomeration of the cellulose particles. Ultimately, nanometer-sized particles were not observed following such treatment. Finally, products exhibited limited thermal stability due to the introduction of sulfone groups into the cellulose structure during the initial acid treatment. The change in the cellulose color was observed during the drying process, while preparing the material for SEM analysis confirmed this effect. The preliminary treatment of cellulose described in this paper, consisting of hybrid acid hydrolysis or oxidation in combination with medium-frequency sonication, clearly indicates the direction of further research on the reduction of the size of cellulose fibers. The novelty of this study was the demonstration of the beneficial effects of oxidation treatment and sonication. As a result, the dimensions of the cross-section of the fibers were reduced. Therefore, avoiding the agglomeration of the fibers, which was the case with the acid treatment. The hybrid method described herein based on the oxidation and sonication of Södra Black R or microcrystalline cellulose, could potentially be used for the in situ generation of cellulose nanofilms, for example, for the preservation or reinforcement of wooden or paper antique items. The authors still believe that the hybrid (i.e., chemical-physical or biological-physical) treatment of cellulose can give satisfactory results in terms of obtaining nanocellulose, which is why further research in the cellulose pre-treatment should focus on optimizing the chemical oxidizing agent in conjunction with the ultrasound.
